# Effects of COVID-19 vaccination on disease activity in patients with rheumatoid arthritis and psoriatic arthritis on targeted therapy in the COVIDSER study

**DOI:** 10.1136/rmdopen-2022-002936

**Published:** 2023-03-16

**Authors:** José M Álvaro-Gracia, Carlos Sanchez-Piedra, Dante Culqui, Rosa Rosello, Alicia Garcia-Dorta, Cristina Campos, Sara Manrique-Arija, Dolores Ruiz-Montesinos, Inmaculada Ros-Vilamajo, Carlos Rodríguez-Lozano, Mercedes Freire-González, Rafael Caliz, Cristina Bohorquez, Lourdes Mateo Soria, Noemí Busquets, Isabel Castrejon, Fernando Sánchez-Alonso, Enrique González-Dávila, Federico Diaz-Gonzalez

**Affiliations:** 1 Rheumatology Department, Hospital General Universitario Gregorio Maranon, Madrid, Spain; 2 Spanish Agency of Health Technology Assessment, Instituto de Salud Carlos III, Madrid, Spain; 3 Research Unit, Spanish Society of Rheumatology, Madrid, Spain; 4 Rheumatology Department, Hospital General San Jorge, Huesca, Spain; 5 Rheumatology Department, Hospital Universitario de Canarias, La Laguna, Spain; 6 Rheumatology Department, Consorci Hospital General Universitari de Valencia, Valencia, Spain; 7 Rheumatology Department, Hospital Regional Universitario de Málaga, Malaga, Spain; 8 Rheumatology Department, Hospital Universitario Virgen Macarena, Sevilla, Spain; 9 Rheumatology, Hospital Son Llatzer, Palma de Mallorca, Spain; 10 Rheumatology Department, Hospital Universitario Insulsar Gran Canaria Doctor Negrin, Las Palmas de Gran Canaria, Spain; 11 Rheumatology, Complejo Hospitalario Universitario A Coruña Biblioteca, A Coruna, Spain; 12 Rheumatology Department, Hospital Universitario Virgen de las Nieves, Granada, Spain; 13 Rheumatology, Hospital Universitario Príncipe de Asturias, Alcala de Henares, Spain; 14 Rheumatology Department, Hospital Universitari Germans Trias i Pujol, Badalona, Spain; 15 Rheumatology Department, Hospital General de Granollers, Granollers, Spain; 16 Rheumatology, Hospital General Universitario Gregorio Marañón, Madrid, Spain; 17 Research Unit, Instituto de Investigacion Sanitaria Gregorio Maranon, Madrid, Spain; 18 Departamento de Estadística e Investigación Operativa, Universidad de La Laguna, La Laguna, Spain; 19 Department of Internal Medicine, Dermatology and Psychiatry, Universidad de La Laguna, La Laguna, Spain

**Keywords:** COVID-19, Vaccination, Rheumatoid Arthritis, Psoriatic Arthritis

## Abstract

**Objective:**

To investigate the influence of COVID-19 vaccination on disease activity in rheumatoid arthritis (RA) and psoriatic arthritis (PsA) patients under targeted therapies.

**Patients and methods:**

1765 vaccinated patients COVID-19, 1178 (66.7%) with RA and 587 (33.3%) with PsA from the COVID-19 registry in patients with rheumatic diseases (COVIDSER) project, were included. Demographics, disease characteristics, Disease Activity Score in 28 joints (DAS28) and targeted treatments were collected. DAS28-based flare rates and categorised disease activity distribution prevaccination and post vaccination were analysed by log-linear regression and contingency analyses, respectively. The influence of vaccination on DAS28 variation as a continuous measure was evaluated using a random coefficient model.

**Results:**

The distribution of categorised disease activity and flare rates was not significantly modified by vaccination. Log-linear regression showed no significant changes in the rate of flares in the 6-month period after vaccination compared with the same period prior to vaccination in neither patients with RA nor patients with PsA. When DAS28 variations were analysed using random coefficient models, no significant variations in disease activity were detected after vaccination for both groups of patients. However, patients with RA treated with Janus kinase inhibitors (JAK-i) (1) and interleukin-6 inhibitor (IL-6-i) experienced a worsening of disease activity (1.436±0.531, p=0.007, and 1.201±0.550, p=0.029, respectively) in comparison with those treated with tumour necrosis factor inhibitor (TNF-i). Similarly, patients with PsA treated with interleukin-12/23 inhibitor (IL-12/23-i) showed a worsening of disease activity (4.476±1.906, p=0.019) compared with those treated with TNF-i.

**Conclusion:**

COVID-19 vaccination was not associated with increased rate of flares in patients with RA and PsA. However, a potential increase in disease activity in patients with RA treated with JAK-i and IL-6-i and in patients with PsA treated with IL-12/23-i warrants further investigation.

**WHAT IS ALREADY KNOWN ON THIS TOPIC**

- A potential relationship between COVID-19 vaccination and disease flares in rheumatoid arthritis (RA) or psoriatic arthritis (PsA) is still under debate. Some case series-based studies, most of them using questionnaires or proxies, have not detected an association between the two conditions. However, some case reports have been published showing disease flare-ups after COVID-19 vaccination. In addition, there is a paucity of data from large registries that systematically analyse changes in clinical activity using a combined score as the Disease Activity Score in 28 joints in patients with RA and PsA after COVID-19 vaccination.

**WHAT THIS STUDY ADDS**

- In this study, we have analysed data from a well-established national registry in which disease activity in patients with RA and PsA was systematically assessed before and after COVID-19 vaccination. Data provide reassurance about the lack of flaring effect of COVID-19 vaccination in patients with RA and PsA treated with targeted therapies. However, using a random coefficient model, we detected an association between increased disease activity and COVID-19 vaccination in patients treated with Janus kinase inhibitor, interleukin-6 inhibitor or interleukin-12/23 inhibitor, compared with those treated with tumour necrosis factor inhibitor.

**HOW THIS STUDY MIGHT AFFECT RESEARCH, PRACTICE OR POLICY**

- Confirmation of these results may have implications for the follow-up of patients, especially if the current trend towards revaccination against COVID-19 continues.

## Introduction

Vaccines are a cornerstone of public health and have proven to be essential to the prevention of many serious infectious diseases for more than a century.^1^ However, in the context of patients with autoimmune inflammatory rheumatic diseases and those treated with medications that modulate the immune system, vaccination has raised some questions about efficacy, immunogenicity and safety.^2^

As the COVID-19 vaccination programme progresses worldwide, one of the questions that has been raised is whether patients with autoimmune rheumatic diseases and on immunomodulatory therapy would respond adequately in terms of safety and immunogenicity to COVID-19 vaccines. Current evidence supports that both messenger RNA (mRNA)-based and viral vector-based vaccines against SARS-CoV-2 are safe and sufficiently immunogenic in patients with autoimmune inflammatory rheumatic diseases.^3–7^ Vaccination stimulates the immune system, a fact that underlies another important question, which is whether the use of vaccines can cause reactivation of disease in patients with immune-mediated rheumatic disorders. Some studies have recently analysed the effect of COVID-19 vaccination on systemic inflammatory/autoimmune and non-inflammatory rheumatic diseases by assessing flare rates, in most cases via questionnaires^8 9^ or by proxies.^10^ With respect to rheumatoid arthritis (RA), disease flares after COVID-19 vaccination have been also analysed in reports that either focused exclusively on patients with RA^11 12^ or in the context of a broad spectrum of autoimmune and immune-mediated diseases, including patients with RA and psoriatic arthritis (PsA).^5 9 10 13 14^ Although none of these case series-based studies detected an association between COVID-19 vaccination and RA flare-ups, there is a paucity of data on activity changes in patients with RA and PsA from large patient registries that systematically analyse clinical activity using a combined score such as the Disease Activity Score in 28 joints (DAS28).

In the COVIDSER project, the association between COVID-19 vaccination and changes in disease activity in patients with RA and PsA treated with biological disease-modifying antirheumatic drugs (bDMARDs) or targeted synthetic disease-modifying antirheumatic drugs (tsDMARDs) was explored. Data were analysed by both the number of flares and the distribution of categorised disease activity before and after vaccination. To determine the association of different patient and disease characteristics and targeted medications with changes in disease activity, variations in DAS28 values were analysed using a general mixed model with a random intercept and slope over time.

## Patients and methods

### Data source

The COVIDSER study is an observational cohort of patients from three Spanish database registries which included patients with rheumatic diseases: BIOBADASER III, CARMA and RELESSER, all promoted by the Spanish Society of Rheumatology.^15^ In this study, only patients from BIOBADASER III were included. BIOBADASER is a Spanish multicentre observational registry used to assess safety in patients with rheumatic diseases who start treatment with any targeted therapy, including bDMARDs, biosimilars or tsDMARDs. BIOBADASER III is the third stage of this registry, a version developed in December 2015,^16^ which was added to safety outcomes a systematic assessment of effectiveness using commonly accepted disease activity indexes, including DAS28. All patients in BIOBADASER are followed up prospectively, and the recruitment of new patients remains open indefinitely. The information of each patient is added to the registry when he/she initiates bDMARDs/tsDMARDs, every time that a change in bDMARDs/tsDMARDs treatment occurs, and at least once a year for effectiveness issues. To assess consistency and quality, the full database is monitored online annually, and additionally, a sample of patient medical records (10%) is randomly selected and audited annually in situ by a specialised clinical research associate at all 28 participating centres. Further details about the design and operation of the BIOBADASER III registry are available at the BIOBADASER website (biobadaser.ser.es).

### Study design and population

We conducted a prospective observational study of patients diagnosed with RA or PsA included in COVIDSER–BIOBADASER III who were vaccinated with any of the vaccines for COVID-19 available in Spain between 22 April 2021 and 11 December 2021 (n=1765). All patients had received at least two doses of Comirnaty (BioNTech/Pfizer, Mainz, Germany) (1022, 58%) or Spikevax (Moderna Biotech, Cambridge, USA) (255, 14.4%), and one dose of the Vaxzevria (Oxford/AztraZeneca, Nijmegen, Netherlands) (403, 22.8%) or Janssen (Johnson & Johnson, Leiden, Netherlands) (85, 4.8%) vaccines, all of which were considered to be complete COVID-19 vaccination regimens by the end of 2021.

### Outcome variables

The following data were collected: (1) patient information, including sex, date of birth, diagnosis, and date of RA or PsA diagnosis; (2) data on DMARDs treatment, types, and duration of bDMARD/tsDMARD prior to and at the time of vaccination; (3) data on the relevant COVID-19 vaccine, type and number of injections; and (4) disease activity as DAS28–erythrocyte sedimentation rate (hereinafter DAS28)^17^ at every visit, both as continuous values and categorised into four groups: remission (DAS28 <2.6), low (DAS28 2.6 (3.2)), moderate (DAS28 3.2 (5.1)) and high (DAS28 >5.1) disease activities.^18^ The closest available DAS28 to vaccination in the prevaccination period and the furthest available DAS28 after COVID-19 vaccination were chosen for analysing changes in categorised disease activity. A disease flare was defined as a DAS28 increase of >1.2 between two consecutive visits.^11 18^

Treatment modifications, such as delaying or skipping methotrexate or leflunomide prior to either vaccine dose, were not documented in the registry. Treatment with bDMARDs/tsDMARDs prior to vaccination was maintained in all patients in whom a postvaccination activity assessment was available.

### Statistical analysis

Data are summarised as relative frequencies for categorical variables, means±SD for normally distributed variables, except when indicated, and median (IQR P25–P75) for non-normal data. Comparisons were performed using the Pearson χ^2^ test, Kruskal-Wallis test or Student t-test according to the type of variable and the number of groups to be compared. Comparisons of the proportions of disease activity in a categorised manner before and after vaccination were performed with the McNemar test for paired samples. To avoid any possible seasonal effect in the analysis of the flare rates, a generalised linear model with Poisson distribution and logarithmic link function (log-linear regression) was used to compare the number of flares between the 6-month period after vaccination with both the 6-month period immediately prior to vaccination and with the same 6 months of the previous year. Random coefficient models with both random intercepts and random slopes were constructed to determine the effect of patient characteristics and targeted medication on DAS28 as continuous measures at baseline and their annual rate of change in the prevaccination and postvaccination periods. The model is described as follows:

$$Y_{ij}=\alpha_{i}+\beta_{i}^{j}t_{j}+\varepsilon_{ij}$$

being

$$\alpha_{i}=\alpha +\delta_{1}1X_{1i}+\cdots +\delta_{1k}X_{ki}+\alpha_{i}$$

$$\beta_{i}^{j}=\left\{ \begin{array}{ll} \beta^{pV}+\delta_{21}X_{1i}+\cdots +\delta_{2k}X_{ki}+b_{i}^{pV} & ift_{j}<0\\ \begin{array}{c} \beta^{Vm}+\delta_{31}X_{1i}+\cdots +\delta_{3k}X_{ki}+b_{i}^{Vm}\\ \beta^{V}+\delta_{41}X_{1i}+\cdots +\delta_{4k}X_{ki}+b_{i}^{V} \end{array} & ift_{j}\geq 0 \end{array}\right.$$

where $Y_{ij}$ represents the values of DAS28 of patient *i* at time *j*; $t_{j}$ is the time given in years with reference to the vaccination date, being equal to 0 at the time of vaccination and negative or positive before and after vaccination, respectively; $X_{hi}$ ( h=1,…,k) is the value of the covariate or factor *h* in the patient *i*; $\alpha_{i}$ is the independent term or intercept of patient *i* (behaviour of DAS28 at time 0, ie, at the time of vaccination) and with $a_{i}∼N\left(0,\sigma_{a}\right)$ as its random component; $\beta_{i}^{j}$ is the slope of the evolution of patient *i* in the prevaccine ( $t_{j}§amp;lt;0)$ ) and postvaccine $\left(t_{j}\geq 0\right)$ periods, the latter depending on the type of vaccine administered (*Vm* (mRNA-based) or *V* (viral vector-based)), representing the annual rate of change of DAS28, with $b_{i}^{pV}∼N\left(0,\sigma_{b}^{pV}\right)$ , $b_{i}^{Vm}∼N\left(0,\sigma_{b}^{Vm}\right)$ and $b_{i}^{V}∼N\left(0,\sigma_{b}^{V}\right)$ being their respective random components and, α , $\delta_{lh}$ (for *l*=1, 2, 3 and y *h*=1,…, k), $\beta^{pV}$ , $\beta^{Vm}$ and $\beta^{V}$ being the coefficients of the model to be estimated. To avoid the effect of disease activity before the biological on the trend of the DAS28 variation, the first observation period was not included in the random coefficient model. The covariates used were age, time of disease evolution, family of bDMARDs or tsDMARDs, and number of previous drug families used, all of them at the time of vaccination. Since age at vaccination, time of disease evolution and the number of families of previous biologics showed significant correlations (all with r>0.3 and p<0.001), to avoid collinearity, only the age at vaccination, which showed a lower Akaike information criterion corrected, was included in the model.

## Results

A total of 1765 patients, 1178 (66.7%) with RA and 587 (33.3%) with PsA, were included in this study. The baseline demographics characteristics, type of targeted therapy used at the time of receiving a COVID-19 vaccine and the different types of vaccines used are shown in table 1. The total median disease duration was 11.1 (IQR 5.9–18.0) years. All patients included in this study were on targeted therapy at the time of COVID-19 vaccination, mostly (50.3%) on tumour necrosis factor inhibitor (TNF-i) (44% of patients with RA and 62.2% of patients with PsA), followed by Janus kinase inhibitor (JAK-i) in 18.2% of patients with RA and interleukin-17 inhibitor (IL-17-i) in 20.2% of patients with PsA. The highest percentage of patients were undergoing their first targeted therapy (1109, 62.8%). Table 1 also shows the disease activity, as assessed by DAS28, with 50.8% and 60% of patients with RA and PsA, respectively, in remission (DAS28 <2.6) or exhibiting low disease activity (DAS28 2.6–3.2).

**Table 1:** Demographic characteristics, disease activity, COVID-19 vaccines and biological treatment of patients included in the study, categorised by diagnosis and in total

	Rheumatoid arthritis (n=1178)	Psoriatic arthritis (n=587)	P value	Total (N=1765)
Female, n (%)	926 (78.6)	318 (54.2)	<0.001	1244 (70.5)
Race, n (%)			0.002	
Caucasian	1104 (93.7)	572 (97.3)		1675 (94.9)
Latin American	51 (4.3)	7 (1.2)		58 (3.3)
Others	23 (2.0)	9 (1.5)		32 (1.8)
Age (years)	60.5±12.2	55.3±11.6	<0.001	58.7±12.2
Age, n (%)			<0.001	
< 45	137 (11.6)	116 (19.8)		253 (14.3)
(45–65)	633 (53.7)	345 (58.8)		978 (55.4)
(65–75)	268 (22.8)	105 (17.8)		373 (21.2)
≥75	140 (11.9)	21 (3.6)		161 (9.1)
Rheumatic disease duration (years), median (IQR)	11.9 (6.4–18.9)	9.7 (5.1–15.9)	<0.001	11.1 (5.9–18.0)
Time from the first biological (years). median (IQR)	4.6 (1.5–11.2)	4.5 (2.0–8.4)	0.532	4.5 (1.7–10.5)
Vaccine type			0.317	
Pfizer	665 (56.4)	357 (60.8)		1022 (58.0)
AstraZeneca	281 (23.9)	122 (20.8)		403 (22.8)
Moderna	176 (14.9)	79 (13.5)		255 (14.4)
Janssen	56 (4.8)	29 (4.9)		85 (4.8)
Disease activity				
DAS28*	3.33±1.47	3.00±1.45	<0.001	3.22±1.47
DAS28 categorised, n (%)			<0.001	
Remission (< 2.6)	410 (34.8)	263 (44.8)		673 (38.1)
Low activity (2.6–3.2)	188 (16.0)	89 (15.2)		277 (15.7)
Moderate activity (3.2–5.1)	416 (35.3)	178 (30.3)		594 (33.7)
High activity ≥5.1	164 (13.9)	57 (9.7)		221 (12.5)
Biologics at vaccination				888 (50.3)
TNF-i	523 (44.4)	365 (62.2)		888 (50.3)
IL-17-i	–	118 (20.1)		120 (6.8)
Anti-CD20	99 (8.4)	–		100 (5.7)
IL-6-i	190 (16.1)	–		193 (10.9)
T cell activation-i	147 (12.5)	–		148 (8.4)
IL-12/23-i	–	57 (9.7)		57 (3.2)
PDE4-i	–	27 (4.6)		27 (1.5)
JAK-i	214 (18.2)	15 (2.6)		229 (13.0)
Number of biologics prior to vaccination			0.109	
1	844 (71.6)	424 (72.2)		1268 (71.8)
2	200 (17.0)	99 (16.9)		299 (16.9)
3	74 (6.3)	47 (8.0)		121 (6.9)
4 or more	60 (5.1)	17 (2.9)		77 (4.4)

*Prevaccination values.

IL-6-i, interleukin-6 inhibitor; IL-17-i, interleukin-17 inhibitor; IL-12/23-i, interleukin-12/23 inhibitor; JAK-i, Janus kinase inhibitor; PDE4-i, phosphodiesterase-4 inhibitor; T-cell activation-i, T-cell activation inhibitor; TNF-i, tumour necrosis factor inhibitor.

**Figure 1:**
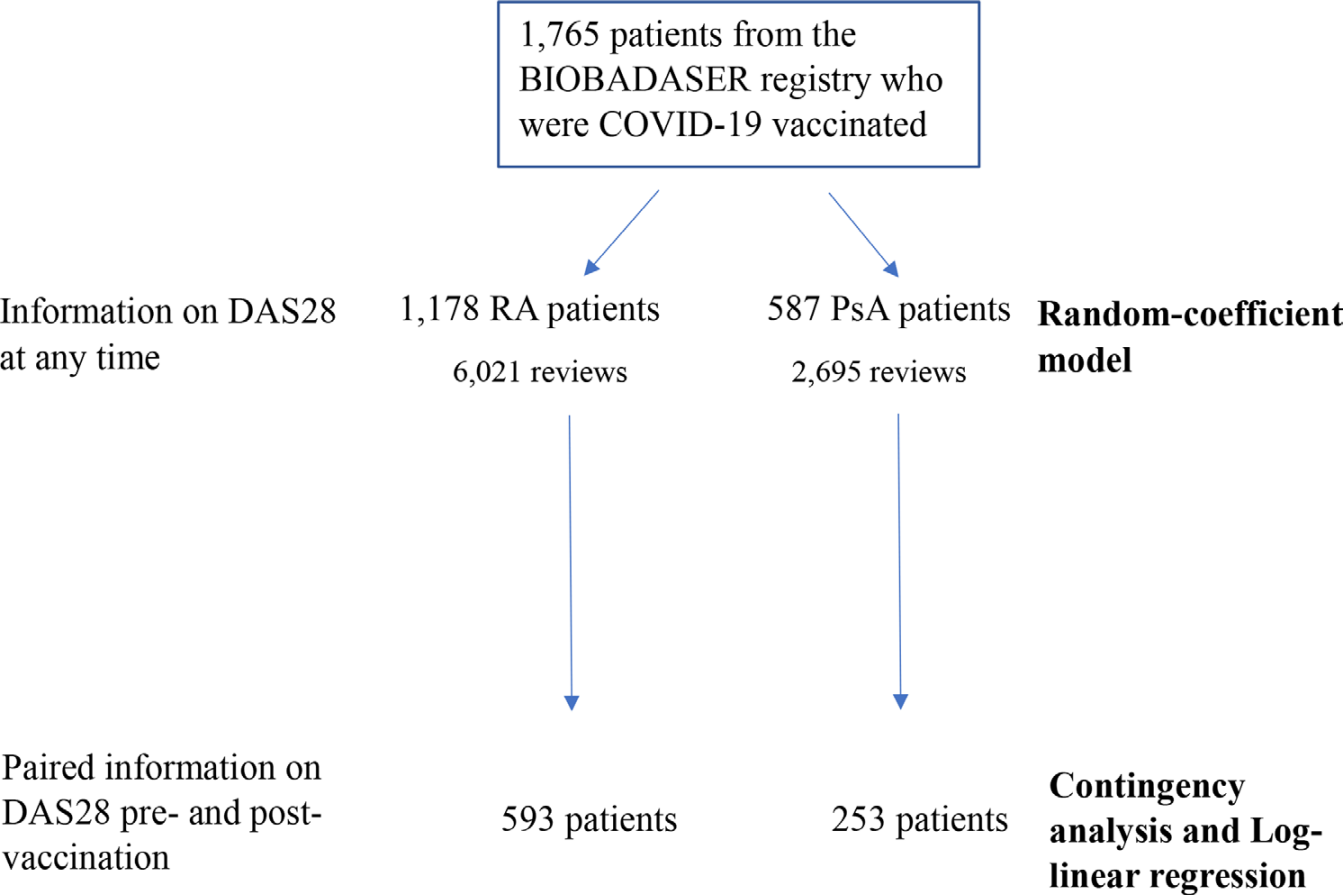
Flowchart showing the number of patients and number of reviews used for each type of statistical analysis (in bold). DAS28, Disease Activity Score in 28 joints; PsA, psoriatic arthritis; RA, rheumatoid arthritis.

Overall, there was a median postvaccination follow-up time of 90 (IQR 43–122) days, 90 (IQR 43–129) days for patients with RA and 79 (IQR 39–118) days for patients with PsA. Disease activity, as assessed by DAS28, was collected at least once during postvaccination follow-up in 731 out of 1178 (62.0%) patients with RA and in 310 out of 587 (58.8%) patients with PsA. Of these, 593 patients with RA and 253 PsA had disease activity paired data before and after vaccination (figure 1).

### Rheumatic disease activity and COVID-19 vaccination

#### Rheumatoid arthritis

In our first approach, we analysed disease activity in patients with RA categorised as remission, low, moderate or high activity^18^ before and after vaccination. Table 2 shows the contingency analysis of disease activity in patients with RA who had at least one assessment of disease activity before and after vaccination (n=593). The prevaccination percentage distribution of the four DAS28-based activity categories did not significantly change after COVID-19 vaccination (p=0.319). Similarly, a non-significant difference in the distribution of categorised disease activity before and after vaccination was observed when the patients receiving TNF-i (p=0.522) or other targeted compounds (p=0.644) were analysed separately at the time of vaccination (data not shown).

**Table 2:** Contingency analysis of categorised disease activity by DAS28 in patients with RA and PsA pre-COVID-19* and post-COVID-19† vaccination

Categorised disease activity	Rheumatoid arthritis, n (%)			Psoriatic arthritis, n (%)		
	Prevaccination	Post vaccination	P value‡	Prevaccination	Post vaccination	P value‡
Remission (< 2.6)	225 (37.9)	251 (42.3)	0.319	120 (47.4)	129 (51.0)	0.681
Low (2.6–3.2)	104 (17.5)	106 (17.9)		44 (17.4)	42 (16.6)	
Moderate (3.2–5.1)	201 (33.9)	186 (31.4)		70 (27.7)	69 (27.3)	
High activity ≥5.1	63 (10.6)	50 (8.4)		19 (7.5)	13 (5.1)	
Total patients	593	593		253	253	

*The most recent available DAS28 evaluation prior to the date of vaccination (−7.68±6.48 months).

†The most recent available DAS28 evaluation at 3 months post vaccination (2.64±1.68 months).

‡McNemar's test for paired samples.

DAS28, Disease Activity Score in 28 joints.

When disease flares were analysed in patients with RA, the annual rate of flares (number of flares with respect to the total number of patients during each period) varied from 11.2% to 18.8% (mean±SD 14.2±3%) during the 9 years prior to vaccination (data not shown). This was similar to that observed during the 6-month period before and after vaccination: 12.6% and 14.2%, respectively (table 3). Log-linear regression showed that there was no significant difference in the rate of flares in the 6-month period after vaccination compared with the rate of flares in the previous 6 months (OR 1.14, 95% CI 0.83 to 1.56, p=0.424). To rule out any seasonality effect, the rate of flares in the 6-month period after vaccination was also compared with the same 6 months of the previous year. Similarly, there was a non-significant trend towards an increase in the flare rate in the postvaccination period (OR 1.42, 95% CI 0.94 to 2.13, p=0.129).

**Table 3:** Percentage of flares during the pre-COVID-19 and post-COVID-19 vaccination periods

	Follow-up time (years or months)	Rheumatoid arthritis			Psoriatic arthritis		
		Patients (n)*	Flares (n)	%	Patients (n)*	Flares (n)	%
Prevaccination	1	712	104	14.6	354	45	12.7
	From 12th month to 6th month prior to vaccination	317	32	10.1	166	19	11.4
	From 6th month to vaccination date	570	72	12.6	264	27	10.2
	From 6th month to 3rd month prior to vaccination	199	26	13.0	83	10	12.0
	From 3rd month to vaccination date	359	46	12.8	172	17	9.8
Post vaccination	At 6 months post vaccination	593	84	14.2	253	25	9.9
	From time to vaccination to 3rd month	410	52	12.7	178	18	10.1
	From 3rd to 6th month post vaccination	231	32	14.2	86	7	8.1

Thick line indicates the time of COVID-19 vaccination.

*Number of patients in each period who had at least one prior review.

**Figure 2:**
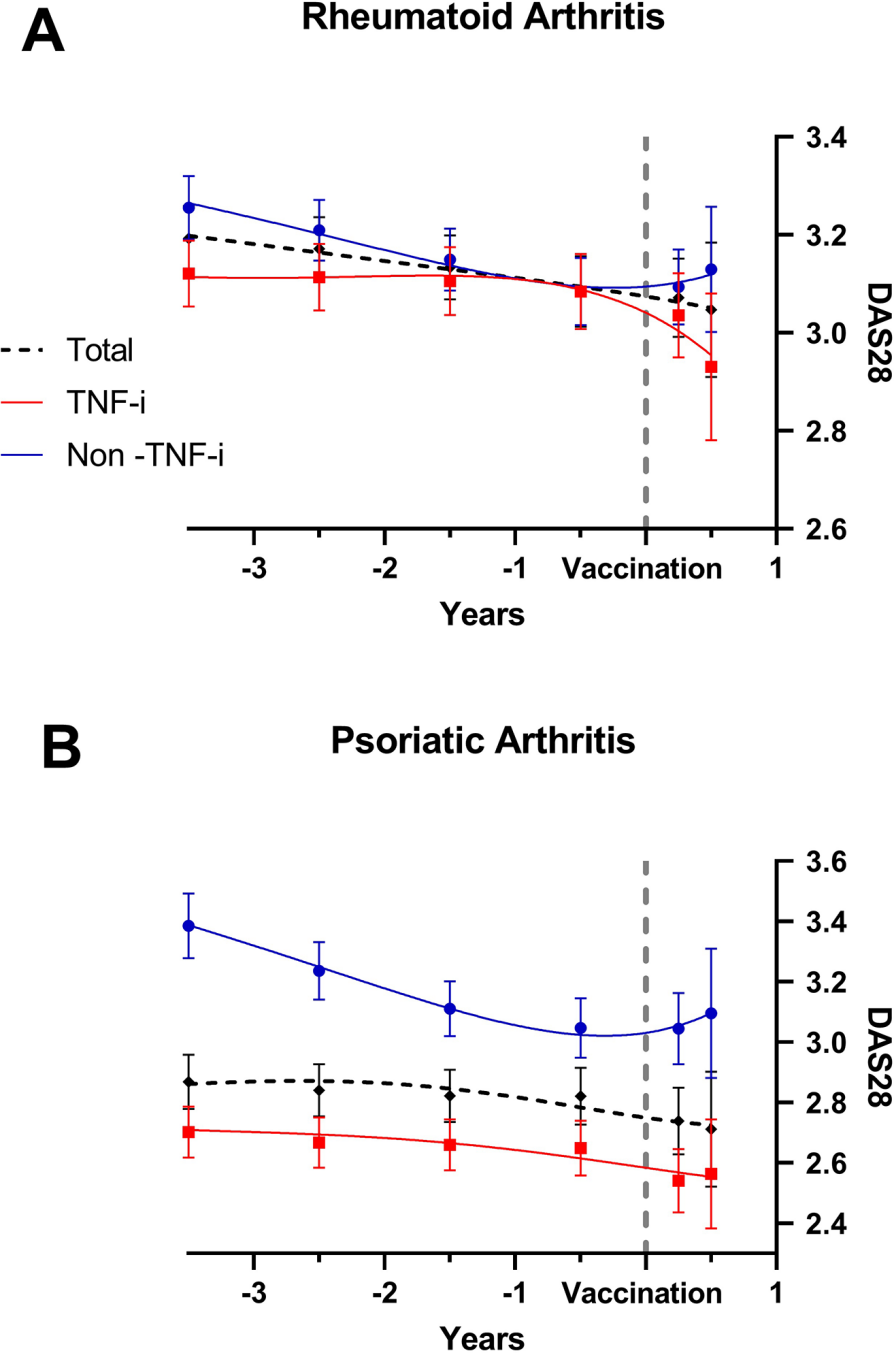
XY scatter plot showing the annual rate of change of DAS28 in the random coefficient model before and after COVID-19 vaccination in patients with (A) RA and (B) PsA in the total population and separated into TNF-i treated and non-TNF-i treated. Numbers represent mean±SE. DAS28, Disease Activity Score in 28 joints; PsA, psoriatic arthritis; RA, rheumatoid arthritis; TNF-i, tumour necrosis factor inhibitor.

To get further insight into the effect of COVID-19 vaccination on disease activity, we then studied its relationship with DAS28 assessed as a continuous variable. Figure 2A shows the variation of DAS28 in the 3 years before and 6 months after COVID-19 vaccination analysed as the total population as well as patients on TNF-i or other non-TNF-i-targeted therapies. No significant effects of vaccination were detected in either group of treatment. Table 4 shows the effect of different covariates on DAS28 assessed as a continuous measure at the time of vaccination (baseline), as well as the annual rate of change over the prevaccination and postvaccination periods. During the prevaccination period, DAS28 revealed an annual variation similar between the two genders and not associated with the age at vaccination. In this prevaccination period, non-TNF-i-targeted therapy showed statistically significant, although very modest, activity-reducing effects on DAS28 with respect to TNF-i (−0.052±0.015, p<0.001). When these changes were analysed over time following COVID-19 vaccination (postvaccination period), no significant associations were observed between age at vaccination and type of targeted therapy with respect to TNF-i, with men showing a significantly worse evolution of disease activity than women with respect to the annual rate of change of DAS28 (0.939±0.464, p=0.043) (table 4). When the effect on DAS28 was analysed by targeted therapy family (anti-B-cell, IL-6-i, T-cell co-stimulation-i and JAK-i compounds) in the postvaccination period, we found no significant changes except in patients treated with JAK-i and IL-6-i who showed a significant worsening of disease activity (1.436±0.531, p=0.007, and 1.201±0.550, p=0.029, respectively) in comparison with those treated with TNF-i (online supplemental table 1).

10.1136/rmdopen-2022-002936.supp1 Supplementary data

**Table 4:** Effects of patient characteristics and targeted therapy on baseline DAS28 and on annual rate of changes in DAS28 prevaccination and post vaccination in patients with RA

	Effects on baseline DAS28 (points)		Effects on annual rate of change in DAS28 (points/year)			
		P value	Prevaccination	P value	Post vaccination	P value
Intercept	2.600±0.211	<0.001	−0.038±0.042	0.366	−0.871±1.019	0.393
Gender (ref. woman)	−0.361±0.099	<0.001	−0.026±0.019	0.172	0.939±0.464	0.043
Age at vaccination (per year)	0.010±0.003	0.004	0.001±0.001	0.252	0.004±0.016	0.800
Targeted therapy at vaccination (ref. TNF-i)	−0.066±0.068	0.332	−0.052±0.015	<0.001	0.581±0.372	0.118

Plus-minus values represent means±SE.

DAS28, Disease Activity Score in 28 joints; RA, rheumatoid arthritis; ref., reference; TNF-i, tumour necrosis factor inhibitor.

#### Psoriatic arthritis

The effect of COVID-19 vaccination on PsA activity was analysed in a similar manner as that of RA. Table 2 shows the contingency analysis of categorised disease activity of patients with PsA who had been evaluated during the prevaccination and postvaccination periods (n=253). As with patients with RA, the percentage distribution of patients with PsA in the four disease activity categories was similar before and after vaccination (p=0.681). The same analysis separating by the use of TNF-i (p=0.378) or other targeted compounds (p=0.490) also showed no significant differences before and after COVID-19 vaccination (data not shown). The annual percentage of flares in patients with PsA ranged from 10.3% to 13.4% (mean±SD 11.7±1) during the 9-year prevaccination period, similar to what was observed when we analysed the 6-month period before and after vaccination, 10.2% and 9.9%, respectively. The flare rate showed a non-significant variation in the 6 months post vaccination compared with both the previous 6 months (OR 0.93, 95% CI 0.54 to 1.60, p=0.797) and the same period in the previous year (OR 1.25, 95% CI 0.83 to 2.02, p=0.365).

As in the RA population, we then studied the relation between COVID-19 vaccination and disease activity as measured by DAS28 as a continuous variable. No significant changes in DAS28 were detected prior to or after vaccination in the global population or in the TNF-i-treated and non-TNF-i-treated groups (figure 2B). Sex or age did not influence the annual change of DAS28 in the prevaccination or postvaccination period (table 5). In the prevaccination period, as observed in patients with RA, patients with PsA treated with non-TNF-i-targeted therapy showed a modest, although statistically significant, reduction in DAS28 with respect to those treated with TNF-i (−0.107±0.031, p<0.001) (table 5). When these changes were analysed over the postvaccination period, no differences in DAS28 were detected in patients with PsA treated with TNF-i-targeted or non-TNF-i-targeted therapies. We then analysed the effect on DAS28 by targeted therapy families (IL-17-i, interleukin-12/23 inhibitor (IL-12/23-i), PDE4-i, JAK-i and others) in the postvaccination period. No significant changes in DAS28 were observed except in patients treated with IL-12/23-i who showed a significant worsening of disease activity (4.476±1.906, p=0.019) compared with those treated with TNF-i (online supplemental table 2).

10.1136/rmdopen-2022-002936.supp2 Supplementary data

**Table 5:** Effects of patient characteristics and targeted therapy on baseline DAS28 and on annual rate of change in DAS28 both prevaccination and post vaccination in patients with PsA

	Effects on baseline DAS28 (points)		Effects on annual rate of change in DAS28 (points/year)			
		P value	Prevaccination	P value	Post vaccination	P value
Intercept	2.644±0.279	<0.001	0.029±0.059	0.620	−1.748±1.366	0.201
Gender (ref. woman)	−0.790±0.105	<0.001	0.002±0.021	0.922	0.050±0.512	0.922
Age at vaccination (per year)	0.007±0.005	0.115	−0.001±0.001	0.376	0.025±0.023	0.275
Targeted therapy at vaccination (ref. TNF-i)	0.302±0.091	0.001	−0.107±0.031	<0.001	0.617±0.549	0.261

*Plus-minus values represent means±SE.

PsA, psoriatic arthritis; ref., reference; TNF-i, tumour necrosis factor inhibitor.

## Discussion

The most important findings of this study can be summarised as follows: (1) in patients with RA and PsA treated with targeted therapy, COVID-19 vaccination is not associated with disease worsening, as assessed by categorised disease activity or arthritis flares; (2) however, when DAS28 variations were analysed as a continuous measure using a predictive model, patients treated with JAK-i or IL-6-i in RA and with IL-12/23-i in PsA showed greater disease activity as measured by DAS28 than those treated with TNF-i after COVID-19 vaccination.

A review conducted by the European Alliance of Rheumatology Associations prior to the COVID-19 pandemic concluded that most protein-based vaccines have shown effectiveness in patients with autoimmune rheumatic diseases, including RA, and that vaccination, although limited by insufficient statistical potency in most studies, does not appear to cause an increase in underlying rheumatic disease activity.^2^ However, as vaccination against SARS-CoV-2 has progressed worldwide, subjective symptoms have reportedly become exacerbated in approximately 10%–13% of patients with rheumatic diseases soon after vaccination.^8^ While traditional vaccine technology, including inactivated, protein-based and live attenuated vaccines, has been used for decades, the most widely used vaccines for COVID-19 are based on the SARS-CoV-2 modified spike protein mRNA delivered via lipid nanoparticles. The lack of experience with this novel approach for generating immunity, in addition to initial reports of postmarketing cases of arthritis reactivation^19 20^ or new onset of rheumatic diseases after vaccination,^21 22^ has driven concerns about whether COVID-19 vaccination may trigger flares in vulnerable populations, including patients with RA and PsA.

Our study, which involved a considerable number of patients who are systemically monitored via a national registry, showed that patients with RA and PsA treated with targeted therapies did not experience a significant worsening in the rate of arthritis flare-ups after a median follow-up of 3 months after COVID-19 vaccination. In our study, the seasonal effect on arthritis flares^23^ was eliminated by comparing the flare rate after COVID-19 vaccination not only with that of the immediately preceding period but also with that of the same months of the previous year. With respect to flares, our finding that there was no increase in the flare rates following COVID-19 vaccination is consistent with previous reports that have focused exclusively on patients with RA^11 12^ or, in the context of a broad spectrum of autoimmune and immune-mediated diseases, which have also studied patients with RA and PsA.^5 9 10 13 14^ All these reports were case series-based, and in most instances, a disease flare was defined using questionnaires or proxies, and in one, a change in treatment was deemed necessary to classify a flare.^14^ Only two of these studies, though they both included a very limited number of patients with RA and PsA, used DAS28 measure to define disease flares.^5 11^

No significant changes were observed in the period after COVID-19 vaccination with respect to the prevaccination period when disease activity was analysed in a categorised manner. This result, consistent with the absence of significant changes in flare rates following COVID-19 vaccination, has not heretofore been reported, as changes in the proportion of categorised disease activity have not been previously used as a method for analysing the effects of COVID-19 vaccination on clinical activity in either of these two diseases.

When evaluating DAS28 as a continuous measure, despite not detecting significant changes in DAS28 following COVID-19 vaccination in patients with RA or PsA, we found some increase in disease activity in patients with RA treated with JAK-i and in patients with PsA treated with IL-12/23-i. This association was independent of age at vaccination or disease duration. To the best of our knowledge, this is the first patient registry-based study to link COVID-19 vaccinations with disease activity assessed by DAS28 as a continuous measure among patients with RA and PsA. The use of a random coefficient model in our study, by allowing the analysis of disease activity at different follow-up periods, offers the possibility of a more detailed analysis of the change in DAS28.

The clinical significance of the increase in DAS28 in patients treated with JAK-i, IL-6-i or IL-12/23-i is probably limited since it was not reflected in the frequency of flares or in changes in DAS28 categorised intervals. However, it raises complex questions on the potential relation between the selective inhibition of cytokine function provided by targeted therapies and the stimulation of the immune system exerted by vaccination.

The literature contains anecdotal reports suggesting a relationship between COVID-19 vaccination and the exacerbation or a new onset of immune/inflammatory disorders such as active CNS demyelination,^24^ myocarditis,^25–27^ thyroidal diseases,^28 29^ interstitial pneumonitis^30^ or allergic dermal reactions.^31^ With respect to chronic inflammatory rheumatic joint diseases, two cases of new-onset persistent polyarthritis have recently been reported in patients who had received an mRNA vaccine. One patient had bilateral pleural effusions with very high serum interferon (IFN)-β levels.^32^ Although a causal relationship between mRNA vaccines and pleural effusion cannot yet be established, the authors suggest that this marked increase in serum IFN-β may reflect an excessive response of the innate immune system to the mRNA vaccine.^32^

The main strength of our study is the use of data from a well-established national registry in which disease activity in patients with RA and PsA was systematically assessed both before and after COVID-19 vaccination and the use of a random coefficient model in our analysis, in contrast with other studies that used data obtained via questionnaires^8 9^ or proxies^10^ and with a significant risk of selection bias. The postvaccination follow-up time was, on average, close to 3 months, a period apparently long enough to detect changes in disease activity in response to vaccination.^5 10–12^ Although no modifications, such as delay or omission of methotrexate or leflunomide prior to any of the vaccine doses, were reported in the database, treatment with bDMARDs/tsDMARDs prior to vaccination was not modified in patients in whom postvaccination assessment of activity was available. This fact reasonably excludes the possibility that the observed effects were due to medication modifications or reflected a limited follow-up period.

One important limitation of this work is that disease activity in patients with PsA was assessed using DAS28. This index was originally developed as an activity index for RA and, although it has been used to assess PsA in previous studies,^33 34^ it has clear limitations particularly in patients with PsA with non-polyarticular or RA-like patterns.^35^ In addition, we cannot exclude the possibility of the existence of transient, self-limiting, flares in our patients that were not detected at the time of disease activity assessment after vaccination. However, we consider that their potential impact on the results is probably limited.

In conclusion, our data provide reassurance about the lack of flaring effect of COVID-19 vaccination in patients with RA and PsA treated with targeted therapies. However, using a random coefficient model, we detected a possible association between increased disease activity and COVID-19 vaccination in patients treated with JAK-i, IL-6-i or IL-12/23-i. This certainly warrants further confirmation using data drawn from other registries, especially if the current trend towards revaccination with COVID-19 vaccines continues.

## Data Availability

Data are available upon reasonable request. Data are available on reasonable request. The data supporting the results of this study are the property of the Spanish Society of Rheumatology (SER) and are not publicly available. However, the data are released upon reasoned request and with the permission of the SER.
