# Supplementary material for: Effects of COVID-19 vaccination on disease activity in patients with rheumatoid arthritis and psoriatic arthritis on targeted therapy in the COVIDSER study
Source: RMD Open. 2023 Mar 16;9(1):e002936. doi: 10.1136/rmdopen-2022-002936 (PMC10030283; doi:10.1136/rmdopen-2022-002936)
Supplement: Supplementary data [file rmdopen-2022-002936supp001.pdf]

Supplementary Table 1. Effects of patient characteristics and targeted therapy on baseline DAS28 and on the annual rate of change in DAS28 pre- and post-COVID-19 vaccination in RA patients*

	Effects on baseline DAS28, points		Effects on annual rate of change in DAS28, points/year			
		P value	Pre-vaccination	P value	Post-vaccination	P value
Intercept	2.611 ± 0.208	<0.001	-0.049 ± 0.042	0.245	-0.742 ± 1.016	0.465
Gender (Ref. woman)	-0.356 ± 0.097	<0.001	-0.020 ± 0.019	0.291	1.083 ± 0.466	0.020
Age at vaccination (per year)	0.010 ± 0.003	0.003	0.001 ± 0.001	0.167	0.001 ± 0.016	0.932
Targeted therapy at vaccination (Ref. TNF-i)						
Anti-B cells	-0.212 ± 0.113	0.061	-0.081 ± 0.023	<0.001	-0.452 ± 0.640	0.480
IL-6-i	-0.353 ± 0.092	<0.001	-0.039 ± 0.019	0.039	1.201 ± 0.550	0.029
T cell coestimulation-i	-0.038 ± 0.108	0.726	-0.124 ± 0.031	<0.001	0.907 ± 0.564	0.108
JAK-i	-0.136 ± 0.109	0.212	-0.350 ± 0.059	<0.001	1.436 ± 0.531	0.007
Others	0.343 ± 0.511	0.502	0.050 ± 0.174	0.775	-0.548 ± 2.684	0.838

*Plus-minus values represent means ± SE

IL-6-i: Interleukin-6 inhibitors; JAK-i: Janus kinase inhibitor; SE: standard error; T cell coestimulation-i: T cell coestimulation inhibitor; TNF-i: tumor necrosis factor inhibitors.
