# Supplementary material for: Effects of COVID-19 vaccination on disease activity in patients with rheumatoid arthritis and psoriatic arthritis on targeted therapy in the COVIDSER study
Source: RMD Open. 2023 Mar 16;9(1):e002936. doi: 10.1136/rmdopen-2022-002936 (PMC10030283; doi:10.1136/rmdopen-2022-002936)
Supplement: Supplementary data [file rmdopen-2022-002936supp002.pdf]

BMJ Publishing Group Limited (BMJ) disclaims all liability and responsibility arising from any reliance placed on this supplemental material which has been supplied by the author(s)

Supplementary Table 2. Effects of patient characteristics and targeted therapy on baseline DAS28 and on the annual rate of change in DAS28, both pre- and post-COVID-19 vaccination, in PsA patients*

	Effects on baseline DAS28, points		Effects on annual rate of change in DAS28, points/year			
		P value	Pre-vaccination	P value	Post-vaccination	P value
Intercept	2.632 ± 0.277	<0.001	0.043 ± 0.059	0.468	-1.569 ± 1.367	0.251
Gender (Ref. woman)	-0.811 ± 0.104	<0.001	-0.009 ± 0.021	0.669	-0.004 ± 0.512	0.994
Age at vaccination (per year)	0.007 ± 0.005	0.124	-0.001 ± 0.001	0.307	0.022 ± 0.023	0.335
Biologic at vaccination (Ref. TNF-i)						
IL-17-i	0.366 ± 0.120	0.002	-0.144 ± 0.050	0.004	0.440 ± 0.637	0.490
IL-12/23-i	0.444 ± 0.150	0.003	0.001 ± 0.046	0.991	4.476 ± 1.906	0.019
PDE4-i	-0.475 ± 0.108	0.726	-0.351 ± 0.114	0.002	2.116 ± 1.549	0.172
JAK-i	0.238 ± 0.273	0.383	-0.353 ± 0.189	0.062	0.965 ± 1.367	0.480
Others	-0.491 ± 0.392	0.211	-0.297 ± 0.077	<0.001	-4.305 ± 3.642	0.237

*Plus-minus values represent means ± SE

DAS28: disease activity score 28; IL-12/23-i: Interleukin-12/23 inhibitor; IL-17-i: Interleukin-17 inhibitors; JAK-i: Janus kinase inhibitor; PDE4-i: phosphodiesterase 4 inhibitor; PsA: psoriatic arthritis; SE: standard error; TNF-i: tumor necrosis factor inhibitors.
